# Combined optical coherence tomography morphologic and fractional flow reserve hemodynamic assessment of non- culprit lesions to better predict adverse event outcomes in diabetes mellitus patients: COMBINE (OCT–FFR) prospective study. Rationale and design

**DOI:** 10.1186/s12933-016-0464-8

**Published:** 2016-10-10

**Authors:** Mark W. Kennedy, Enrico Fabris, Alexander J. Ijsselmuiden, Holger Nef, Sebastian Reith, Javier Escaned, Fernando Alfonso, Niels van Royen, Wojtek Wojakowski, Adam Witkowski, Ciro Indolfi, Jan Paul Ottervanger, Harry Suryapranata, Elvin Kedhi

**Affiliations:** 1 Isala Hartcentrum, Docter van Heesweg 2, Zwolle, The Netherlands; 2 Diagram CRO, Zwolle, The Netherlands; 3 Albert Schweitzer Ziekenhuis, Dordrecht, The Netherlands; 4 University of Giessen, Giessen, Germany; 5 University Hospital of the RWTH Aachen, Aachen, Germany; 6 Hospital Clinico San Carlos, Madrid, Spain; 7 Hospital Universitario de La Princesa, Madrid, Spain; 8 VU University Medical Centre, Amsterdam, The Netherlands; 9 Medical University of Silesia, Katowice, Poland; 10 Institute of Cardiology, Warsaw, Poland; 11 Azienda Ospedaliera Universitaria Materdomini, Catanzaro, Italy; 12 Radboud University Medisch Centrum, Nijmegen, The Netherlands

**Keywords:** Diabetes mellitus, Fractional flow reserve, Major adverse cardiac event

## Abstract

**Background:**

Fractional flow reserve (FFR) is a widely used tool for the identification of ischaemia-generating stenoses and to guide decisions on coronary revascularisation. However, the safety of FFR-based decisions in high-risk subsets, such as patients with Diabetes Mellitus (DM) or vulnerable stenoses presenting thin-cap fibro-atheroma (TCFA), is unknown. This study will examine the impact of optical coherence tomography (OCT) plaque morphological assessment and the identification of TCFA, in combination with FFR to better predict clinical outcomes in DM patients.

**Methods:**

COMBINE (OCT–FFR) is a prospective, multi-centre study investigating the natural history of DM patients with ≥1 angiographically intermediate target lesion in three subgroups of patients; patients with FFR negative lesions without TCFA (group A) and patients with FFR negative lesions with TCFA (group B) as detected by OCT and to compare these two groups with each other, as well as to a third group with FFR-positive, PCI-treated intermediate lesions (group C). The study hypothesis is that DM patients with TCFA (group B) have a worse outcome than those without TCFA (group A) and also when compared to those patients with lesions FFR ≤0.80 who underwent complete revascularisation. The primary endpoint is the incidence of target lesion major adverse cardiac events (MACE); a composite of cardiac death, myocardial infarction or rehospitalisation for unstable/progressive angina in group B *vs.* group A.

**Conclusion:**

COMBINE (OCT–FFR) is the first prospective study to examine whether the addition of OCT plaque morphological evaluation to FFR haemodynamic assessment of intermediate lesions in DM patients will better predict MACE and possibly lead to new revascularisation strategies.

*Trial Registration* Netherlands Trial Register: NTR5376

**Electronic supplementary material:**

The online version of this article (doi:10.1186/s12933-016-0464-8) contains supplementary material, which is available to authorized users.

## Background

Diabetes Mellitus (DM) is associated with more rapidly progressive coronary atherosclerosis and increased mortality compared to non-diabetic patients [1, 2]. Fractional Flow Reserve (FFR) is a pressure-derived intracoronary functional index currently recommended in clinical practice guidelines for stenosis assessment in the absence of objective evidence of myocardial ischaemia [3]. Previous studies have shown that FFR-negative lesions (FFR > 0.80) can be safely treated medically, while FFR-positive lesions (FFR ≤ 0.80) benefit from revascularisation [4, 5]. However, in the majority of the FFR trials performed to date, the percentage of DM patients is low [4–6].

More recently, several studies have suggested that FFR-based revascularisation may not be associated with the same reduction in adverse cardiac events as seen in non-DM patients [7–10], even when the presence of microvascular disease, which might imply ischaemia of non-obstructive origin, is taken into consideration [10]. These findings could be explained by the fact that, in patients with DM, adverse cardiac events after ischaemia-driven revascularisation may be related to the presence of more active atherosclerotic disease, resulting in subsequent acute coronary events or obstructive disease progression, rather than to ischaemic burden at the time of revascularisation [11, 12]. However, this hypothesis of more rapid atherosclerosis progression in high risk plaque, is as yet only postulated.

The PROSPECT study demonstrated an ~12 % 3-year rate of future unanticipated major adverse cardiovascular events (MACE) in non-culprit lesions (NCL) [13]. Furthermore, insulin dependent DM was identified as an independent predictor of NCL MACE, in addition to the presence of thin-cap fibroatheroma (TCFA), minimal lumen area (MLA) ≤4 mm^2^, and a plaque burden ≥70 %. Additionally, the rate of future NCL-related MACE was twice as high among patients with DM hosting at least one thin-cap fibroatheroma TCFA, whereas those DM patients without a TCFA had a more benign prognosis, similar to non-DM patients [14].

Although these NCL’s appeared angiographically mild, significant differences in plaque composition between those patients with versus without DM were noted [15]. Separately, Kato et al. have shown that the incidence of TCFA in patients with DM is higher than in patients without DM [16]. As FFR was not measured in PROSPECT, whether these lesions were truly non-ischaemic is unknown, however given the mild angiographic severity (diameter stenosis; median 36.2 % IQR [31.1, 44.2]) this was unlikely. Nonetheless, it has been shown that these high risk IVUS-detected plaque features have no correlation with FFR functional significance of intermediate coronary lesions, and so whether ischaemia is the only factor in the prediction of future adverse cardiac events is questionable [17].

Several studies using intravascular ultrasound (IVUS), have attempted to address the mismatch between the functional and anatomical significance of intermediate coronary lesions, however these have largely focused on quantitative assessments of MLA, lesion length and plaque burden. From these studies, it has been shown that only a mere moderate correlation between IVUS quantitative measurements and FFR exists [17–19].

Optical Coherence Tomography (OCT) is an intravascular imaging modality that provides a spatial resolution 10 times higher than IVUS [20]. Whilst OCT is superior to IVUS in identifying the haemodynamic significance of coronary stenoses, particularly in vessels <3 mm, its low specificity and only modest diagnostic efficacy precludes its use as a substitute for FFR functional stenosis assessment [21]. However, the true benefit of OCT may centre upon its superior ability to detect high risk vulnerable plaque. With the introduction of OCT, our understanding of plaque morphology and the mechanisms of plaque rupture which result in ACS have been significantly advanced [22, 23]. A thin fibrous cap overlying a lipid-rich necrotic core is believed to be the substrate for most vulnerable plaques, and OCT is the only imaging modality that currently can accurately assess fibrous cap thickness [24]. Multiple intravascular imaging studies have shown the the presence of vulnerable plaque characteristics are more prevalent in DM patients compared to non-DM patients and in particular a greater burden of TCFA lesions [25–28]. Due to these characteristics, OCT is being frequently used to evaluate lesion morphology, however, the predictive value of this modality with regard to future MACE is not well studied.

In the COMBINE (OCT–FFR) study, we propose to investigate the incremental value of OCT plaque morphological evaluation added to FFR haemodynamic assessment of intermediate lesions in DM patients in predicting MACE when following an ischaemia-driven revascularization strategy.

## Study design and objectives

The COMBINE (OCT–FFR) is a prospective multi-centre, international study, involving centres in the Netherlands, Germany, Spain, Italy, Switzerland and Poland. The study was approved by the Institutional Review Board of each participating hospital. The study is registered on the Netherlands Trial Registry under the identifier NTR5376 [29]. 

The primary objective of the COMBINE (OCT–FFR) study is to evaluate whether the presence of certain plaque characteristics considered to carry a high-risk (such as TCFA) in lesions with an intermediate-severe angiographic stenosis but which are non-ischaemic (FFR > 0.80) can predict future adverse cardiac events. For this purpose, we have selected to examine only patients with DM, as these patients constitute the sub-population with the fastest progression of coronary atherosclerosis and where recent evidence suggests that deferred revascularisation based upon FFR assessment may not be as safe as in non-DM patients.

**Fig. 1:**
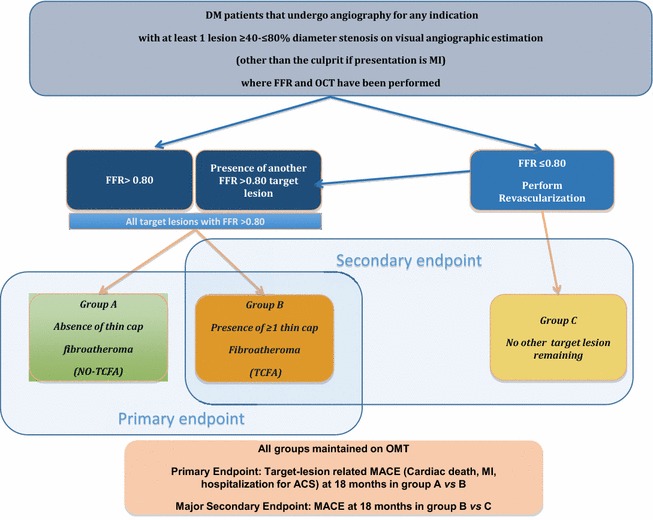
Study flow-chart

The study design is illustrated in Fig. 1. The study population consists of all DM patients with any clinical presentation who undergo FFR assessment in lesions with an intermediate-severe angiographic stenosis. Inclusion and exclusion criteria are presented in Table 1. Lesions which are determined as the culprit lesion in myocardial infarction will not be included, owing to the lack of evidence for FFR in such clinical scenarios. FFR will be performed according to standard protocol using the PressureWire Aeris™ or Certus™ wires, St. Jude Medical, St. Paul, Minnesota, USA. Adenosine is the preferred hyperaemic agent to be administered via the intravenous route at a recommended dose of 140 μg/kg/min to achieve maximum hyperaemia. Should intracoronary adenosine be administered, a dose of 40 μg for the right coronary artery and 80 μg for the left coronary artery is recommended. Once steady-state maximum hyperaemia is achieved, FFR is calculated as the ratio of mean distal intracoronary pressure measured by the pressure wire, and the mean arterial pressure measured through the coronary guiding catheter. In all patients where FFR is performed, OCT assessment using the frequency domain Dragonfly™ OCT system, St. Jude Medical, St. Paul, Minnesota, USA will also take place. Patients with ≥1 lesion which does not undergo revascularisation based upon a FFR negative (>0.80) assessment will be followed clinically. Depending upon the OCT findings, patients without any TCFA (see Additional file 1: Appendix for definition) lesions will form Group A, whilst patients with ≥1 TCFA carrying lesion will form Group B. Finally, patients hosting only FFR-positive target lesions and no other remaining lesions, which have undergone complete index revascularisation either by PCI or CABG will form Group C.

**Table 1:** COMBINE OCT–FFR inclusion and exclusion criteria

Inclusion criteria
Age ≥18 years
History of DM with any indication for angiography (Stable Angina Pectoris or any type of ACS)
At least ≥ 1 de novo target lesion in a native coronary segment with a visually estimated diameter stenosis of between ≥40 and ≤80 %
Exclusion criteria
TIMI flow <3 in the target lesion(s)
Target lesion reference diameter <2.0 mm
Left Ventricular ejection fraction <30 %
Malignancy
Life expectancy <2 years
Unwilling or unable to provide informed consent

*DM* denotes diabetes mellitus; *ACS* denotes acute coronary syndrome; *TIMI* denotes thrombolysis in myocardial infarction

## Study endpoints

### Primary endpoint

The per patient incidence of target lesion(s) related composite MACE defined as: Cardiac death, myocardial infarction (MI), clinically-driven target lesion revascularisation (TLR) or hospitalisation due to unstable or progressive angina at 18 months in the FFR-negative No-TCFA patients (Group A) as compared to the FFR-negative TCFA patients (Group B).

### Major secondary endpoints

1. The per patient incidence of the target lesion(s) related composite MACE: Cardiac Death, MI, clinically-driven TLR or hospitalisation due to unstable or progressive angina between FFR-negative TCFA-positive patients (Group B) and the group of patients with PCI-treated FFR-positive lesions (Group C).
2. The per patient incidence composite MACE: Cardiac death, MI, any clinically-driven revascularisation or hospitalisation due to unstable or progressive angina between FFR- negative TCFA (Group B) and the group of patients with PCI-treated FFR-positive lesions (Group C).
3. The incidence of MACE (Cardiac Death, MI, clinically-driven revascularisation or hospitalisation due to unstable or progressive angina) in patients carrying any non-revascularised TCFA lesion (target or elsewhere within the assessed coronary segments) compared to patients without any identified TCFA lesions.

### Additional secondary endpoints

1. The incidence of the separate components of the primary endpoint at 18 months between Group A and Group B.
2. The incidence of FFR positive (≤0.80) lesions in angiographically mild lesions (<50 % diameter of stenosis) and their rate clinical outcomes at 18 months.
3. The incidence of cardiac death, MI and and clinically-driven target vessel revascularisation (TVR) at 18 months.
4. Clinical predictors of MACE at 18 months in patients with ≥ 1 FFR > 0.80 lesion.
5. Outcome of MACE in older versus younger patients (cut-off 75 years) with ≥ 1 FFR > 0.80 lesion.
6. Impact of HbA1c on the incidence of TCFA and MACE outcomes.
7. The incidence of target lesion related MACE at 18 months in the FFR**-** negative TCFA negative patients (Group A) vs. patients with PCI treated FFR + lesions with no other remaining lesions (Group C).
8. The incidence of target lesion related MACE at 18 months in FFR-negative lesions in patients with versus without ACS at presentation.
9. The impact of renal insufficiency (eGFR < 60 mls/min) on the incidence of TCFA and MACE outcomes.
10. The impact of gender on the incidence of TCFA and MACE outcomes.
11. The per patient incidence of the target lesion(s) related composite MACE defined as Cardiac Death, MI, clinically-driven TLR or hospitalisation due to unstable or progressive angina in the FFR-negative No-TCFA (Group A) and FFR- negative TCFA (Group B) at 3 years (if funding permits).
12. The incidence of a composite endpoint of Cardiac Death, MI, clinically-driven TLR or hospitalisation due to unstable or progressive angina between FFR-negative TCFA (Group B) and the group of patients with PCI treated FFR + lesions with no other remaining lesions (Group C) at 3 years (if funding permits).
13. The impact of MI (prior or at presentation) on the incidence of TCFA and MACE outcomes.
14. The impact of other OCT-detected plaque types (other than TCFA) on the incidence of MACE in Group A and Group B.

## Follow-up data collection and study management

Patient demographics and clinical data at inclusion are collected online in an electronic database (CRO Diagram, Zwolle, The Netherlands). The first 2 patients included in all centres will be fully monitored to identify inconsistent data. Upon discharge and after the intended follow-up period of 18 months, data will be collected at visits at outpatient clinics or, if not feasible, by telephone follow-up and/or a medical questionnaire, carried out by staff who are blinded. Follow-up beyond 18 months is intended, should funding permit. During visits and telephone calls, patients will be interviewed regarding repeat hospitalisations, revascularisation procedures, and myocardial infarction (MI) during follow-up. In case of death, information will be obtained from the patient’s medical chart, local institution and by contact where required with the patient’s cardiologist/general practitioner. FFR and OCT core laboratory will be based at an external independent research centre, in addition to overall trial coordination, data management and study monitoring (CRO Diagram, Zwolle, the Netherlands). An independent clinical events committee will adjudicate all potential clinical endpoints.

Whilst an observational study, without randomisation, in order to confer meaning to the results derived, a power calculation has been made to estimate a projected number of patients that would allow observation of meaningful differences in MACE rates between the above mentioned subgroups of patients. The primary endpoint event rate at 18 months in the A and B group respectively are assumed to be 5 and 20 % respectively. Furthermore, we assume that 1/3 of the patients will have at least one lesion with a positive FFR (≤0.80) as shown from the major FFR trials [4, 5].

An equal distribution of patients between subgroups A and B is expected (50 % in each group) however the power calculation takes in account variations up to 20 % (i.e. an unequal TCFA distribution in both directions up to 30 vs. 70 %). Taking into account an expected loss in follow-up of 7 %, a total of 500 patients enrolled in the study will provide 80 % power to reject the null hypothesis with 5 % type I error. With this number of patients, it is expected that this study is also powered to assess its major secondary endpoint if an equal distribution (in groups A and B) is observed and the target lesion MACE rate in the group C does not exceed 7 % (assumption based on observations from recent novel drug-eluting stent (DES) studies for non-complex lesions [30].

## Statistical analysis

In general, statistics for continuous variables will include mean, median, standard deviation, minimum, maximum, and sample size for each treatment group, and two-sided 95 % confidence intervals of the mean difference between the treatment groups. Binary variables will be described with frequencies, percentages, and two-sided 95 % confidence intervals of the difference in percentages between treatments using exact methods. For time-to-event data, Kaplan–Meier estimates at the indicated time points will be displayed along with 95 % confidence intervals for the difference in the estimates along with log rank test results. In addition, survival curves will be constructed for all time to event secondary endpoints using Kaplan–Meier methods. For the primary endpoint as for the secondary endpoint a multivariate regression analysis will be performed.

## Present status

The COMBINE (OCT–FFR) study started enrolment in April 2015; and as of August 1, 2016, 148 patients have been included. To date 12 participating centres are actively enrolling, and several others are still in the start-up process. End of the enrolment is expected in 2017.

The COMBINE (OCT–FFR) study is investigator driven and is supported by an unrestricted grant from St. Jude Medical. The authors are solely responsible for the design and conduct of this study, all study analyses, and drafting and editing of the manuscript.

## Conclusion

COMBINE (OCT–FFR) is the first large prospective natural history study to examine whether the addition of OCT plaque morphological evaluation and the identification of high-risk plaque features such as TCFA, in combination with FFR haemodynamic assessment in DM patients will better predict MACE and possibly lead to new revascularisation strategies in a group of patients at high risk for future adverse cardiac events.

## Additional file

10.1186/s12933-016-0464-8 **Additional definitions.**

## Abbreviations

DM: diabetes mellitus
FFR: fractional flow reserve
OCT: optical coherence tomography
IVUS: intravascular ultrasound
MACE: major adverse cardiac events
NCL: non-culprit lesion
TCFA: thin cap fibroatheroma
PCI: percutaneous coronary intervention
CABG: coronary artery bypass graft
MI: myocardial infarction
TLR: target lesion revascularisation
