# Supplementary material for: Combined optical coherence tomography morphologic and fractional flow reserve hemodynamic assessment of non- culprit lesions to better predict adverse event outcomes in diabetes mellitus patients: COMBINE (OCT–FFR) prospective study. Rationale and design
Source: Cardiovasc Diabetol. 2016 Oct 10;15:144. doi: 10.1186/s12933-016-0464-8 (PMC5057218; doi:10.1186/s12933-016-0464-8)
Supplement: Supplementary file 1 — 10.1186/s12933-016-0464-8 Additional definitions. [file 12933_2016_464_MOESM1_ESM.docx]

**Appendix:**

**Definitions**

**1. Target Lesion(s) and Culprit lesion(s)**

Refers to any *de novo* lesion with an angiographic visual estimation of ≥ 40%- ≤ 80% diameter stenosis (DS) that is located in a non-grafted coronary segment. In patients with an MI at presentation the *target lesion* should be different from the *culprit* lesion. Culprit lesions should be determined based on angiography and 12-lead ECG, however if in MI patients a culprit lesion cannot be established than OCT can be used in adjunction to 12-lead ECG and angiographic findings to determine the most plausible culprit lesion (ruptured atherosclerotic plaque with superimposed fresh thrombus). All the other lesions that fulfil the above mentioned angiographic criteria could be considered as target lesion even if a ruptured plaque (but not judged as culprit) is observed. In patients presenting with stable angina pectoris (SAP) or unstable angina pectoris (UAP), all lesions that fulfil angiographic criteria can be considered as target lesion independently from the OCT findings.

**2 Thin-cap FibroAtheroma (TCFA) lesion**

Any lesion with predominantly lipid rich plaque which in the thinnest part of the atheroma cap measures ≤ 65μm on OCT assessment. Calcification nodules may be present but the plaque should be predominantly lipid rich and the thinnest part of the cap should not be localized completely over a calcium nodule.

**3 Death**

Is defined as death of any cause.

**3.1 Cardiac death**

Is defined as any sudden death, death related to acute myocardial infarction, arrhythmia or congestive heart failure, death secondary to a cerebrovascular accident, or death directly related to PCI or CABG, even if the ultimate cause of death is not clearly a cardiac event (e.g., infection).

**3.2 Non-cardiac death**

Is any death which is specifically non-cardiac in ethology.

**4 Myocardial infarction**

**4.1 Spontaneous myocardial infarction**

Detection of rise and/or fall of cardiac biomarkers (CKMB or troponin) with at least one value above the 99th percentile of the upper reference limit (URL) together with evidence of myocardial ischaemia with at least one of the following:

- Symptoms of ischemia

- ECG changes indicative of new ischaemia [new STT changes or new, persistent, non-rate related left bundle branch block (LBBB)]

- Development of pathological Q waves (≥ 0.03 seconds in duration or ≥ 1mm in depth) in ≥ 2 or more contiguous precordial leads or ≥ 2 adjacent limb leads of the ECG

- Imaging evidence of new loss of viable myocardium or new regional wall motion

abnormality

**4.2 PCI-related MI (<72 hours after procedure)**

Elevation of the cardiac troponin value > 10 x 99th percentile of the URL in patients with a normal baseline reference level or an increase of > 20%, if the baseline values are elevated, but are stable or falling. In addition, at least one of the following: (i) new pathologic Q waves or new left bundle branch block (ii) angiographic documented new graft or new native coronary occlusion (iii) imaging demonstration of new loss of viable myocardium or new regional wall motion abnormalities is required.

**5 Revascularisation**

**5.1 Clinically indicated**

(i) A revascularisation is clinically indicated if angiography shows a DS ≥ 50% (QCA) and if one of the following occurs:

(1)  A positive history of recurrent angina pectoris presumably related to the target vessel.

(2)  Objective signs of ischaemia at rest (ECG changes) or during exercise test (or equivalent) presumably related to the target vessel.

*Requires documented decision to re-intervene based on clinical symptoms and/or results of non-invasive functional testing, before any coronary imaging.*

(ii) Abnormal results of any invasive functional diagnostic test (e.g. Doppler flow velocity reserve, fractional flow reserve) independently from symptoms and degree of angiographic stenosis.

(iii) Presence of a ruptured coronary atherosclerotic lesion with or without adjacent thrombus OCT/IVUS evaluation on follow-up in presence of clinical symptoms that can be judged related to a ACS. The results of these test in (ii) and (iii) must be documented in the Case Report Form.

**5.2 Not Clinically indicated**

Are re-interventions for:

1.All stenoses <50% (diameter stenosis by QCA) in the presence or absence of ischaemic  signs or symptoms that do not fulfil criteria in point (ii) and (iii).

2.All stenoses ≥50% (diameter stenosis by QCA) without ischaemic signs or symptoms and  do not fulfil criteria in point ii and iii.

**6 Hospitalisation due to unstable or progressive angina**

Any re-hospitalisation due to unstable angina or progressive angina according to the Canadian Cardio-vascular Society Angina Classification (CCS) class III-IV leading to revascularisation

**7 Diabetes Mellitus**

Active treatment with insulin or an oral hypoglycaemic agent on admission. For patients diagnosed with diabetes who are on dietary therapy alone, documentation of an abnormal fasting blood glucose (>7 mmol/l), blood glucose > 11.1 mmol/l at any time, or abnormal glucose tolerance test based on the World Health Organization criteria is required.

**8 Smoking Status**

Smoker: regular cigarette smoking in the prior 6 months

Non-smoker: no cigarette smoking at any time (according to WHO also includes former smokers who have quit smoking for at least 10 years).

Former smoker: those who had quit smoking at least 6 months before the index PCI.

**9 Family History of Premature Coronary Artery Disease (CAD)**

Myocardial infarction, angiographic documentation of CAD or sudden abrupt death without obvious cause, before the age of 55 in a first-degree blood male relative (parent, sibling, or children related by blood) or before the age of 65 in a first-degree blood female relative.

**10 History of Hypercholesterolemia**

Patients with any one of the following: 1. Prior total cholesterol > 6.21 mmol/l 2. Prior or present treatment with a lipid lowering agent

**11 Arterial Hypertension**

Arterial hypertension is considered to be present when a person's systolic blood pressure is 140 mmHg or greater, and/or their diastolic blood pressure is 90 mmHg or greater on 2 different occasions, or active treatment with antihypertensive drugs.

**12 Angina Assessment**

The angina status of the patient will be assessed according to the Canadian Cardiovascular Society (CCS) Classification at all follow up contacts (calls or visits).
